# Cytotoxic CD8+ T Cells Expressing CXCR5 Are Detectable in HIV-1 Elite Controllers After Prolonged *In Vitro* Peptide Stimulation

**DOI:** 10.3389/fimmu.2020.622343

**Published:** 2021-02-24

**Authors:** Philipp Adams, Gilles Iserentant, Jean-Yves Servais, Linos Vandekerckhove, Guido Vanham, Carole Seguin-Devaux, the PhenoCure Study Group

**Affiliations:** ^1^ Department of Infection and Immunity, Luxembourg Institute of Health, Esch-sur-Alzette, Luxembourg; ^2^ Departments of Biomedical and Clinical Sciences, Institute of Tropical Medicine, Antwerp, Belgium; ^3^ Department of Biomedical Sciences, University of Antwerp, Antwerp, Belgium; ^4^ Department of Internal Medicine, Ghent University, Ghent, Belgium

**Keywords:** HIV controllers, functional cure, viral suppressive capacity, multifunctional CD8^+^ T cells, CXCR5 CD8 T cells + +, elite controllers

## Abstract

Antiretroviral therapy (ART) is not curative as HIV-1 persists in long-lived viral reservoirs. Consequently, patients are dependent on life-long drug adherence with possible side effects. To overcome these limitations strategies of a functional cure aim at ART free viral remission. In this study, we sought to identify detailed subsets of anti-viral CD8^+^ T cell immunity linked to natural long-term control of HIV-1 infection. Here, we analyzed HIV controllers and ART suppressed progressors for *in vitro* viral suppressive capacity (VSC) at baseline and after peptide stimulation. Functional properties and phenotypes of CD8^+^ T cells were assessed by IFN-γ ELISPOT and 18 color flow cytometry. HIV controllers showed significantly increased suppression at baseline as well as after peptide stimulation. IFN-γ secretion and the proliferation marker Ki67 positively correlated with VSC. Moreover, the detailed phenotype of three distinct multifunctional memory CD8^+^ T cell subsets were specific traits of HIV controllers of which two correlated convincingly with VSC. Our results underline the importance of multifunctional CD8^+^ T cell responses during natural control. Especially the role of CXCR5 expressing cytotoxic subsets emphasizes potential surveillance in sites of reservoir persistence and demand further study.

## Introduction

Antiretroviral therapy (ART) halts the progression to AIDS by suppressing viral replication but is not curative, because HIV-1 persists in long-lived latent reservoirs (1–3). Interestingly a small group of HIV-1 infected patients succeeds in maintaining low plasma viral loads (pVL) over many years in the absence of therapy. They are regarded as the role model for a functional cure, which refers to ART free HIV-1 remission. HIV controllers are subdivided according to their level of viremia into elite controllers (EC), with pVL < 20 copies, and viremic controllers (VC), with pVL ≤ 2000 copies (4). The association of EC with certain HLA-I alleles is one of the strongest indications for an involvement of anti-viral CD8^+^ T cells (5–7). An abundance of literature has characterized the anti-HIV-1 CD8 + T cell response in EC displaying greater cytotoxicity (8, 9), higher proliferation (10, 11) and polyfunctionality (12, 13). There is strong evidence that specificity, quality and broadness are all key determinants of efficient HIV-1- specific CD8^+^ T-cell mediated responses (14). Also do a number of functional studies substantiate that protection against HIV-1 is more related to the quality (such as polyfunctionality) of the T cell response than to its magnitude (15). Moreover higher frequencies of early T cell responses preferentially targeting the Gag protein are predictive of low viral set point, control of infection and found to be dominant in EC (16–18). Among other aspects, targeting Gag constraints immune escape as it comes with high costs in viral fitness when mutated (19, 20). Therefore eliciting polyclonal T cell responses targeting conserved epitopes in Gag is a major objective in HIV vaccinology (21).

HIV controllers are a heterogeneous population. Potent anti-HIV-1 CD8^+^ T cell responses are not restricted to HIV controllers carrying protective HLA alleles. Moreover very weak HIV-1-specific T cell responses can be found in HIV controllers while non-HIV controllers may present with such favorable genetic background (22, 23). Taken together, these finding indicate that additional factors may contribute to achieve sustained viral control (24).

Direct evidence for viral suppressive capacity (VSC) *in vitro* is derived from the viral inhibition assay (VIA), which measures viral outgrowth from CD4^+^ T cells in the presence of CD8^+^ T cells. Several studies highlighted improved VSC in EC compared to progressors (9, 25, 26). The breadth of Gag CD8^+^ T cell responses was found to be associated with higher VSC and reduced viral loads *in vivo* (27, 28). Interestingly low VSC predicts CD4^+^ T cell decline in untreated patients (29). We recently reported that VSC after peptide stimulation was correlated in ART-treated patients with higher expression of immune checkpoint markers on subsets of terminally differentiated effector memory (TEMRA) CD8^+^ T cells producing higher level of IFN-γ, TNF-α and IL-10 (30). However to understand which specific CD8^+^ T-cell subsets are most effective in viral suppression of HIV controllers is still needed.

Over the last years secondary lymphoid organs were pinpointed as the major anatomical sites of HIV persistence during ART (31). Recent studies highlighted the importance of lymph node resident follicular CD4 ^+^ T helper cells for ongoing viral replication correlating with the presence CD8^+^ T cell (32). Further data from SIV infection suggest a functional role of B-cell follicle homing CD8^+^ T cells (CXCR5^+^) that could contribute to control SIV infection (33). To what degree CXCR5^+^CD8^+^ T cells are present in EC and which functional phenotype they have remains to be clarified.

To that aim, we performed an in-depth characterization of CD8^+^ T cells after expansion of Gag-specific memory responses. We identified three distinct multifunctional subsets in natural controllers associated with strong CD8^+^ T-cell responses and VSC. For the first time, we report the emergence of CXCR5 cytotoxic subsets from peripheral blood of EC that might target the productive HIV-1 reservoir.

## Material and Methods

### Study Design and Participants

A total of 56 patients were recruited in the Belgian clinical AIDS Reference Centers of Antwerp (Institute of tropical medicine), Brussels (Centre Hospitalière Universitaire Sint Pierre and Universitair Ziekenhuis Brussels), Liège (Centre Hospitalière Universitaire) and Hasselt (Jessa Ziekenhuis). Inclusion criteria defined elite controller (EC) as being treatment naïve, pVL ≤ 20 copies/ml with a duration ≥ 12 months; viral controller (VC) as treatment naïve, pVL 20–2000 copies/ml with a duration ≥ 12 months; ART as patients under treatment, pVL ≤ 20 copies/ml for ≥ 24 months. The numbers of patients in the different categories were as follows: EC (n=15), VC (n=11), ART (n=30). Although not set as a inclusion criterium it is worth to mention that all patients had stable CD4^+^ T cell counts above 500 cells/mm^3^. Patients signed informed consent for longitudinal blood donation of 100 ml every 6 months over a period of 3 years. The study was approved by each local ethical committee and received the ethical approval from the university hospital of Antwerp (Belgium) under the registration number: B300201731330 as a multicenter Belgian cohort called as PhenoCure. Furthermore, anonymized blood samples from healthy donors (HD) (seronegative for HIV, HBV, and HCV) were collected from the red cross in Luxembourg as controls for certain immune assays.

Blood was processed within a maximum of 6 h to maintain functionality of PBMCs. Whole blood separation was done by gradient centrifugation using lymphoprep™ (Stemcell technologies) and LeucoSEP™ (Greiner Bio-One) tubes according to harmonized standard operating procedures. Isolated PBMCs were cryopreserved in commercially available medium (Cryostor, CS10, Stemcell technologies, Vancouver, Canada) and stored in liquid nitrogen (-196°C). Plasma and whole blood samples were stored at -80°C.

### Viral Inhibition Assay

The viral inhibition assay was set-up as described previously with significant modifications (34). CD4^+^ T cells and CD8^+^ T cells were prepared and isolated from freshly thawed PBMCs as follows:

Preparation of CD4^+^ T target cells: freshly thawed PBMCs were stimulated with an anti-human CD3/CD8 bi-specific monoclonal antibody (1µg/ml, NIH AIDS Reagent Program) and cultured during 7 days in “R10/IL-2” medium, composed of Rosewell Park Memorial Institute (RPMI) medium with 10% fetal bovine serum (FBS), L-Glutamine (0,4 mM per ml), supplemented with Penicillin and Streptomycin at 100 IU/ml and interleukin-2 (IL-2 500 IU/ml, Gentaur, Kampenhout, Belgium). PBMCs were cultured at a density of 2x10^6^ cells per ml at 37°C with 5% CO_2_. Fresh R10/IL-2 medium was added on day 3 and 5 of culture. On day 7 cells were harvested, washed three times with cold PBS (Gibco, Fisher Scientific, Waltham, Massachusetts, USA) and CD4^+^ T cells were isolated using negative selection magnetic beads (Miltenyi Biotech, San Diego, California, USA) according to the manufacturer’s protocol. Purities of CD4^+^ T cells of more than 90% were confirmed by flow cytometry for CD3 (BUV496, clone OKT3), CD4 (BUV395, clone SK3) and CD8 (BUV737, clone SK1). Enriched cells were washed twice with R10/IL2 and then infected with HIV III_B_ lab strain (NIH AIDS reagent program) at a multiplicity of infection of 10^-3^ using spinoculation at 1200 g for 2 h at 25°C followed by 1 h of incubation at 37°C 5% CO_2_. Cells were washed twice with R10/IL2 and resuspended at 1 x 10^6^cells per ml in R10/IL2 medium to distribute for the VIA culture.

Preparation of CD8^+^ T effector cells: CD8+ T effector cells were prepared in two conditions: stimulated (*in vitro*) and non-stimulated (*ex vivo*). Freshly thawed PBMCs were stimulated with a potential T cell epitope (PTE) Gag peptide pool (NIH reagent program) at 100 ng/ml per peptide in R10 medium and cultured for 7 days at 2 x 10^6^ cells per/ml density without changing the medium. For unstimulated cells freshly thawed PBMCs were cultured overnight in R10 medium at 2 x 10^6^cells per/ml density. Both stimulated and unstimulated PBMCs were harvested on the same day and subjected to CD8^+^ T cell negative selection (Miltenyi Biotech, San Diego, California, USA). Purities of more than 90% were confirmed by flow cytometry as described above. Cells were washed twice with R10/IL2 and resuspended at 1 x 10^6^ cells per ml in R10/IL2 medium to distribute for the subsequent VIA culture.

The VIA was performed in flat bottom 96-well culture plate (Greiner BIO-ONE) in 200 µl volume of R10/IL2 medium. Each patient sample was run in five conditions and each condition in triplicates. The conditions were, non-stimulated and stimulated CD8^+^ T effector cells in coculture with IIIb-infected CD4^+^ T target cells at an effector-to-target (E:T) ratio of 1:1 and 1:10, as well as IIIb-infected CD4 ^+^ T target cells only. Half of culture medium was recovered and refreshed every 3 days. Specificity of the assay was previously shown (30) and no further group of uninfected controls was included during this study. Levels of p24 antigen in supernatants were quantified on day 14 in coculture by p24 ELISA (BioMARIC, Ghent, Belgium) according to the manufacturer’s protocol. VSC was calculated as log_10_ [p24 CD4^+^ T cells only] – [p24 coculture with CD8^+^ T cells].

### Measurement of Soluble Factors by MULTI-ARRAY ELISA

Several cytokines and chemokines were assessed in plasma of patients and at day 5 of the VIA coculture using two different configurations of MULTI-ARRAY ELISA (mesoscale discovery,Rockville, Maryland, USA) U-plex detection plates respectively. On plasma we assessed IFN-γ, TNF-α, IL1-β, IL-6, IL18, and IP10. On supernatants from the VIA coculture we assessed IFN-γ, IL-2, IL6, IL-12p70, IP-10, TNF-α, TRAIL, CCL4 (MIP1β). Technical procedures were executed according to the manufacturer’s instructions and acquired on the MESO SECTOR S600 (mesoscale discovery) reader.

### ELISPOT for IFN-γ Production

MultiScreen_HTS_-IP, 0,45µm, 96-well plates (Merck Millipore, Burlington, Massachusetts, USA) were coated overnight with anti-human IFN-γ monoclonal antibody (clone 1-D1K, MABTECH, Stockholm, Sweden) diluted 1/100 in PBS at 4°C. The next day plates were washed with PBS and then blocked with R10 medium for 60 min. 2 x 10^5^PBMCs/per well in 200 µl volume were loaded onto the plate supplemented with PTE Gag peptide pool (NIH reagent program) at 100 ng/ml concentration. Samples were run in two conditions: 7 days Gag prestimulated PBMCs and overnight rested non-stimulated PBMCs, each in duplicates for a total duration of 36 h at 37°C 5% CO_2_. Duplicate positive controls were stimulated with PMA (50 ng/ml, Invivogen) and Ionomycin (500 ng/ml, Invivogen). PBMCs from healthy donors were included as to estimate non-specific background signal. After incubation plates were washed with PBS and incubated with biotinylated IFN-γ detection antibody (clone 7-B6-1, MABTECH) diluted 1/100 PBS-0,05 Tween- 0,5% BSA for 2 h. Revelation was performed using HRP Streptavidin for ELISPOT (BD Biosciences) and AEC Substrate Set (BD Biosciences) according to manufacturer’s instructions. Plates were read and analyzed using Easy-Count and the SmartCount (ImmunoSpot, Bonn, Germany) software.

### Flow Cytometry

Detailed cellular phenotyping of PBMCs using 18-color flow cytometry was performed just before the coculture and during coculture (96 h into VIA culture). Before coculture, PBMCs were evaluated in two conditions per patient. Either after 7 days of prestimulation (stimulated condition) or after overnight resting (Non-stimulated condition). Prior to staining both conditions were exposed to PTE Gag peptides at 100 ng/ml per peptide for a total of 16 h with Golgi Stop (1/1000 final dilution, BD Biosciences). Golgi Plug (1/1000 final dilution) was added during the last 10 h of culture together with anti-CD107a antibody (BV421, clone H4A3).

During the VIA coculture, prestimulated CD8+ T cells with infected autologous CD4+ T cells or with non-infected autologous CD4 + T cells at the 1:1 E:T ratio were analyzed after 120 h (5 days) in coculture. Golgi Stop (1/1000 final dilution, BD Biosciences), Golgi Plug (1/1000 final dilution) and anti-CD107a antibody (BV421, clone H4A3) were added into the culture 6 h before staining.

Cells were further harvested for antibody staining, washed twice with 4 ml of FACS buffer (PBS with 1%BSA) and stained with the LIVE/DEAD Fixable NearIR Dead cell stain kit (Thermo Fisher Scientific) as well as the antibodies for the extracellular antigens for 30 min at 4°C. Subsequently cells were washed twice with 4 ml FACS buffer and permeabilized/fixed using the FOXP3 transcription factor staining buffer set (eBioscience, Santa Clara, California, USA) according to the manufacturer’s instructions. Next the intracellular antigens were stained for 30 min. Cells were washed twice with 4 ml of FACS Buffer and acquired on the Fortessa LSR flow cytometer (BD Biosciences, USA).

Different panels were used for phenotyping sharing a common backbone panel for lineage gating on T cells containing anti human antibodies for CD4 (BUV395, clone SK3, BD), CD3 (BUV496, clone UCHT1), CD8 (BUV737, clone SK1), CD45RA (BV460, clone HI100), CXCR5(BV711, clone J52D4), CCR7 (PE-CF594, clone 150305), CD14 (PECy5, clone G1D3), and LIVE/DEAD Fixable NearIR Dead cell stain kit (APC-Cy7, Thermo Fisher Scientific). The additional antibodies used in the panels were anti-human: HLA-DR (BV510, clone L243), IL2 (BV605, clone MQ1-17H12), CD57 (BV605, clone NK-1) IFN-γ (BV650, clone 4S.B3), PD-1 (BV786, clone EH12.1), Ki67 (Alexa Fluor 488, clone B56), CD38 (PERCP-Cy5, clone HIT2), TNF-α (APC, clone Mab11), IL-2 (PE, clone MQ1-17H12), Granzyme B (PE, clone GB11), Perforin (PE-Cy7, clone B-D48), MIP-1β (APC, clone D21-1351).

### Quantification of HIV-1 DNA

Cell-associated HIV-1 DNA was isolated from purified CD4^+^ T cells (CD4^+^ T cell microbeads, Miltenyi) from final quantities of 2x10^6^ cells per patient. Extraction was done using the DNeasy Blood and tissue kit (Qiagen, Hilden, Germany) according to manufacturer’s instruction. Absolute quantification of HIV-1 DNA was performed by droplet digital PCR (ddPCR) (Bio-Rad, Hercules, California) as previously described (35) with total HIV-1 DNA primers and probes as reported (36).

### Data Analysis and Statistics

Flow cytometry data were analyzed using Flow Jo (version 10, BD Biosciences) and Kaluza (version 1.5, Beckman Coulter). Analysis was done by a classical gating strategy focused hierarchically on lymphocytes, single cells, live-cells, CD3^+^, CD8^+^ cells. Subsequently CD8^+^ T cells were subdivided into the four main subsets (Naïve (CD45RA^+^CCR7^+^), Central Memory (CM) (CD45RA^-^CCR7^+^), Effector memory (EM) (CD45RA^-^CCR7^-^) and terminally differentiated effector memory subsets re-expressing CD45RA (TEMRA) (CD45RA^+^CCR7^-^). Subsequently each subset was analyzed using a Boolean gating strategy combining eight phenotypic and functional markers. Data was exported as.csv files and imported into the Tableau (Tableau Software, Seattle, Washington, USA) environment for data cleaning and organization. Statistical testing was executed with the QluCore (Qlucore, Scheelvägen, Sweden) software using Kruskal Wallis non-parametric comparison with a false discovery rate (FDR) of 0.2. Identified hits were exported into Prism version 8 (GraphPad Software, California, USA) and represented with from non-parametric multiple comparison with Dunn’s correction.

All other data was analyzed with Prism (version 8, GraphPad) with non-parametric statistical methods tests (specified in figure legends for each data set).

## Results

### Participants Demographics, Plasma Markers, and Cell Associated HIV-1 DNA

The characteristics of HIV-1 patients recruited in the PhenoCure cohort are given in **Table 1**. Inclusion criteria were mentioned in the *Material and Methods* section defining the three categories. Importantly, EC and VC were treatment naïve with the exception of two female EC which have been ART-treated during pregnancy. Information on the specific subtype of HIV-1 was not available for all patients. With regard to plasma markers an elevated concentration of circulating IL-18 in ART patients [median 534,8 pg/ml, (290,9–1478)] compared to EC [336 pg/ml, (149,1–412,1)] was observed (**Supplementary Figure 1A**), suggesting that systemic inflammation stays elevated during ART and in contrast is more limited during natural control of HIV-1 infection. The other cytokines (IL-6, IP-10, TNF-α) didn’t differ among patient groups (**Supplementary Figures 1B–D**).

**Table 1:** Patient characteristics recruited in the PhenoCure cohort.

Category	All	EC	VC	ART
Number of individuals (n)	**56**	15	11	30
Median **Age** (years)	**48**	50	44	49
Range	**27–69**	34–69	27–65	28–68
Median **CD4 counts** (cells/µL)	**863**	937	936	778
Range	**542–1980**	712–1450	566–1980	542–1487
Median **CD4 nadir** (cells/µL)	**552**	665	647	299
Range	**63–848**	231–848	340–748	63–801
Median **current pVL** (copies/ml)	**>20**	< 20	86	< 20
Range	**19–1280**	/	19–1280	/
Median **highest pVL recorded** prior to inclusion (copies/ml)	**16800**	< 20	182	469750
Range	**19–10000000**	19-11400	54–47800	5470 - 10000000
Median **HIV-1 DNA** (copies/million CD4)	**1013**	124	185	3680
Range	**46–11146**	46–285	68–9648	208–11146
**Sex** (percentage of male participants)	**74%**	40%	45%	80%

All, all participants of the cohort; EC, elite controllers; VC, viral controllers; ART, ART treated patients. Age (lifetime of participants in years); CD4 counts (Number of CD4+ T cells); CD4 nadir (lowest CD4 T cell count recorded); current pVL (plasma viral load at the time of analysis); highest pVL recorded (highest plasma viral load recorded prior to inclusion into the cohort); HIV-1 DNA (total cell associated HIV-1 DNA in CD4+ T cells); Sex (sex of participants).

Cell-associated total HIV-1 DNA was significantly lower for EC [median 124 copies/million CD4 + T cells, (46–2530)] compared to the ART group [median 3680 copies/million CD4 + T cells, (208–11146)] whereas the values for VC (median 185 copies/million CD4 + T cells, [68-9648]) were not significantly different between EC and ART-treated patient (**Supplementary Figure 2A**). These findings are concordant to published literature (37).

### Viral Suppressive Capacity Significantly Increases by Gag Stimulation and Was More Pronounced During Natural Control of HIV-1 Infection

**Figure 1:**
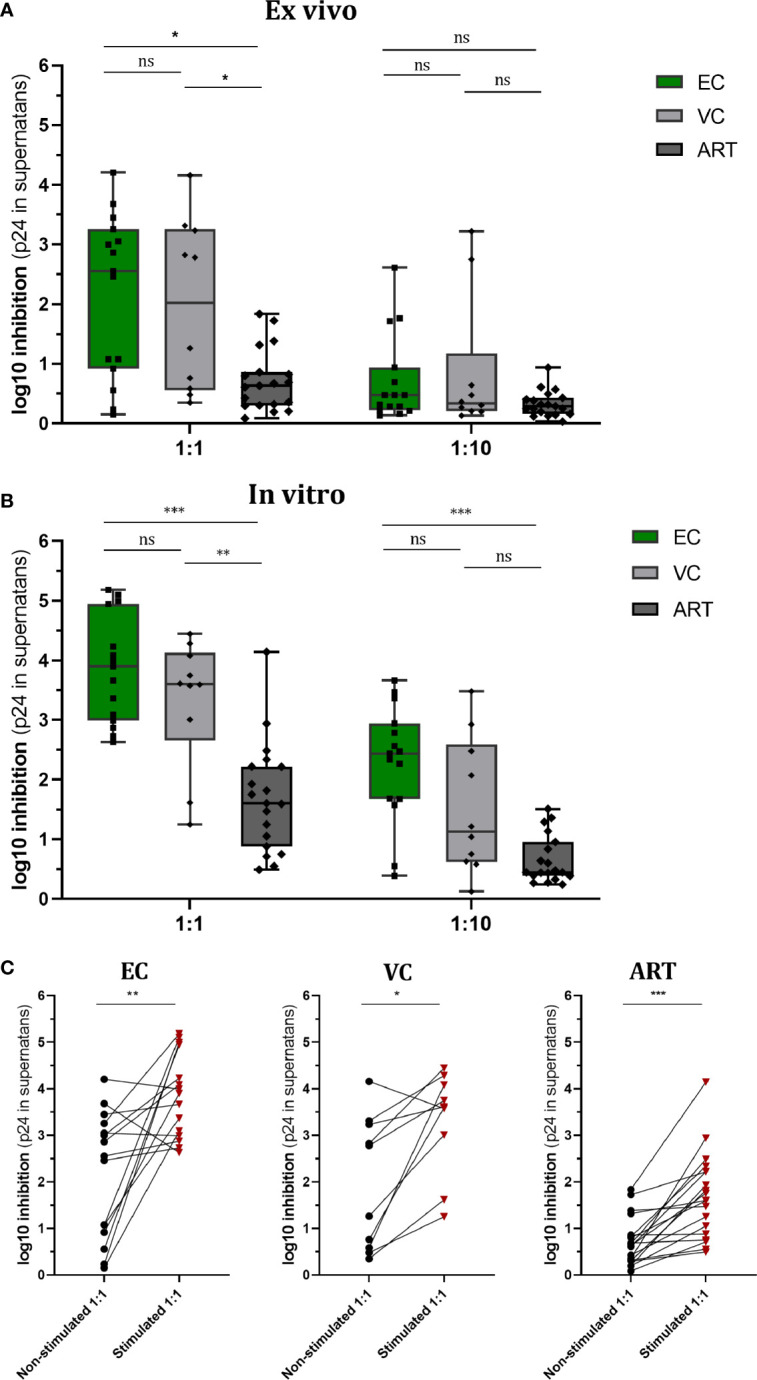
Viral Suppressive capacity is highest in EC and significantly increased after peptide stimulation for all patient groups. Log_10_ inhibition of HIV p24 production measured for non-stimulated **(A)** and Gag PTE peptide pool (NIH) stimulated **(B)** CD8+ T cells towards superinfected autologous CD4+ T cells at E/T of 1:1 and 1:10. Statistical testing by non-parametric Kruskal Wallis with Dunn’s correction for multiple comparison. VSC compared between non-stimulated and stimulated conditions for each patient group **(C)**. Analysis by Mann Whitney non-parametric statistics. *<0.05, **<0.01, ***<0.001; ns: not significant.

Increased VSC is commonly observed during *in vivo* viral control. In the present study, two questions were addressed: First, to what extent will pre-stimulation with Gag peptides improve VSC of CD8^+^ T cells compared to non-stimulated, baseline VSC. Secondly, how this potential induction of VSC compares among different clinical phenotypes, namely HIV controllers (EC and VC) and treated progressors (ART). In addition we ran VIA assays using two E:T ratios (1:1 and 1:10) to estimate the per cell capacity of suppression. In the non-stimulated (*ex vivo*) setting VSC in 1:1 ratios was significantly increased for EC and VC as compared to ART (median VSC in log_10_: EC 2.5, VC 2.02, ART 0.63). When the E:T ratio was reduced (1:10), VSC was decreased so that no further difference among groups was observed (**Figure 1A**) (median VSC in log_10_: EC 0.47, VC 0.33, ART 0.29).

After Gag PTE stimulation (*in vitro*) for 7 days, VSC in EC and VC was still significantly higher than in ART patients (median VSC: EC 3.9, VC 3.6, ART 1.6.). Interestingly VSC stayed significantly elevated for EC when ten times less effector cells were present (**Figure 1B**) (median VSC: EC 2.4, VC 1.1, ART 0.4). This points to a higher presence of HIV-1 specific T cells or an increased per cell based ability to suppress viral outgrowth. Interestingly stimulation with the Gag peptides enhanced VSC in all patient groups (**Figure 1C**). The ratio of enhancement for VSC stimulated over non-stimulated condition (EC. 1.56, VC 1.78, ART 2.54) was the highest for ART patients.

Collectively, these data underline the importance of anti-Gag CD8^+^ T cell response for viral suppression across different clinical phenotypes. EC and VC harbored a high VSC already at baseline which further increased upon stimulation. ART patients, did show significant increase upon pre-stimulation as well raising the question which aspects of T cell immunity delineate progressors from controllers in this condition.

### IFN-γ Secretion Increases After Stimulation and Correlates Moderately With VSC

**Figure 2:**
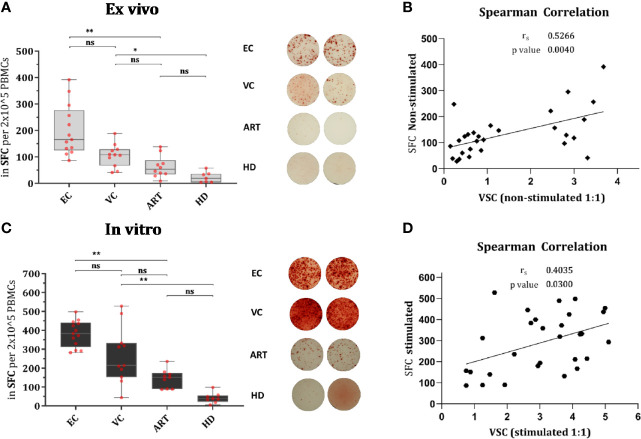
IFN-γ secretion is significantly increased in EC and correlates moderately with VSC. Overnight rested PBMCs were either ex vivo (Non-stimulated) **(A)** or *in vitro* (Stimulated) with Gag PTE peptides for 7 days **(C)** before IFN-γ secretion of PBMCs was measured by ELISPOT during 36 h in culture. Data plotted as spot forming counts (SFC) and representative duplicates are shown as photos. Statistical analysis by Kruskal Wallis nonparametric statistics with correction for multiple comparison by Dunn’s method. All HIV-1 infected patients were analyzed for correlation with VSC by Spearman’s rank correlation for Non-stimulated **(B)** and Stimulated **(D)** PBMCs. *lt;0.05, **<0.01; ns: not significant.

IFN-γ secretion, measured by ELISPOT, is a hallmark of antiviral immunity and is frequently used to assess the effect of T cell vaccination strategies in clinical trials. In order to know whether secretion of IFN-γ does correlate with VSC we assessed the number of spot-forming cells (SFC) in non-stimulated PBMCs and Gag PTE-stimulated PBMCs. In both conditions, HD PBMCs showed minimal responses, as expected; ART patients showed a weak response as well (not significantly different from HD), but enhanced by stimulation (**Figure 2A**). In EC the response was heterogeneous but significantly increased from VC and ART when PBMCs were not-stimulated (**Figure 2A**). After stimulation for 7 days ELISPOT responses increased in all patient groups as compared to their respective non-stimulated condition (Wilcoxon paired rank test, EC: p < 0.0005, VC: p < 0.005 and ART: p< 0.005). The fold change of stimulated versus non-stimulated (ratio of median values) condition per patient group was: EC (2.25), VC (1,98), ART (2.84). Hence, the number of IFN-γ secreting cells increased most in ART patients. However EC had still the highest total measures after peptide stimulation for 7 days (median SFC, EC: 392, VC: 214, ART: 151). Overall, the number of IFN-y secreting cells did correlate moderately with VSC for both conditions (**Figures 2B, D**).

### Upon Gag Peptide Stimulation CD8^+^ T Cells Increase Ki67 and CXCR5 but IFN-γ and CD107a Are Significantly Upregulated in HIV Controllers Only

**Figure 3:**
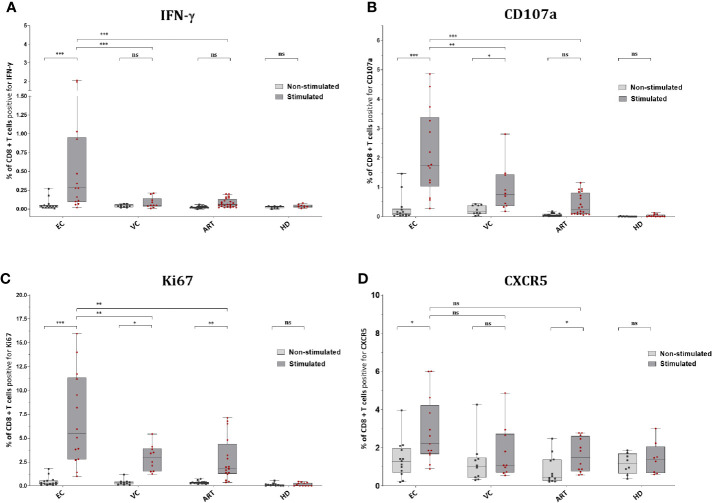
CD8^+^ T cells of EC express significantly more IFN-γ, CD107a, Ki67 and CXCR5 after peptide stimulation. Expression of IFN-y **(A)**, CD107a **(B)**, Ki67 **(C),** and CXCR5 **(D)** on CD8^+^ T cells *ex vivo* or Non-stimulated (light grey) and *in vitro* or Stimulated (dark grey). Statistical comparison by Kruskal Wallis with Dunn’s correction for multiple comparison across groups. Paired Wilcoxon rank test comparison of non-stimulated versus stimulated conditions within groups. *<0.05, **<0.01, ***<0.001; ns: not significant.

EC showed significantly increased production of IFN-γ in CD8^+^ T cells after Gag PTE prestimulation compared with non-stimulated PBMCs but also compared to other patient groups (**Figure 3A**). Remarkably, pre-stimulation selectively enhanced IFN-γ production in EC and not in VC or ART (**Figure 3A**). CD107a expression was similarly low in non-stimulated cells from all groups but significantly increased in EC and VC after pre-stimulation, although more pronounced in EC than in VC or ART patients (**Figure 3B**). A similar trend was observed for Ki67, a proliferation-associated marker, but the increase in expression after pre-stimulation, was significant in all HIV(+) subjects, including ART. However, EC showed significantly higher percentages of Ki67 positive cells than VC and ART (**Figure 3C**). Of note Ki67 in the ART group was also significantly increased compared to uninfected, healthy controls after peptide stimulation (p <0.05, not shown in the graph) underlining GAG specific CD8^+^ T cell proliferation. Interestingly an increase in CXCR5 was observed on CD8^+^ T cells which was significant for EC and ART (**Figure 3D**). The analysis of bulk CD8^+^ T cells showed no difference in Granzyme B or Perforin (data not shown). There was no correlation with VSC for IFN-γ, CD107a and CXCR5. Ki67 correlated only weakly with VSC (**Supplementary Figure 3C**). Overall, these data showed that all HIV-1 patients respond to Gag stimulation by upregulation of the proliferation marker, Ki67. IFN-γ and CD107a, however, were selective traits of HIV controllers.

### EC Develop Distinct, Multifunctional, and Cytotoxic CD8^+^ T Cell Subsets Upon Stimulation of Which One Has Potential Access to Lymph Node Follicles

**Figure 4:**
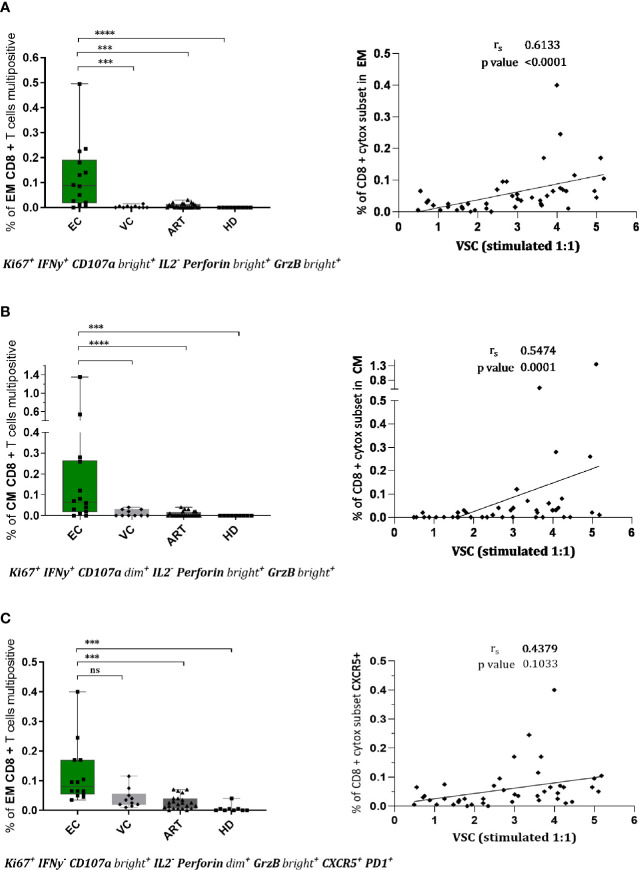
CD8^+^ T cells of EC show multifunctional, cytotoxic profiles in effector and central memory compartment which correlate with viral suppression. Phenotypes of CD8^+^ T cells were assessed upon stimulation for 7 days by flowcytometry. Combinatorial expression of different markers was analyzed by boolean gating strategy. CD8+ effector memory T cells (CCR7-CD45RA-) in **(A, C)** and central memory T cells in **(B)** with respective spearman correlations with viral suppression. The specific combination of expressed proteins is annotated below the graphs. Statistical testing in two steps, first the identification of hits by Kruskal Wallis with a FDR of 0.2 followed by non-parametric Kruskal Wallis comparison with Dunn correction for multiple comparison. ***<0.001, ****<0.0001, ns: not significant.

To assess combined expression of phenotypical and functional proteins of CD8^+^ T cells in detail we established a Boolean gating strategy on the main memory and effector subsets. According to these analyses, we found several rather unique multifunctional subsets in EC (**Figure 4**). Two distinct subsets with a cytotoxic profile expressing Ki67, IFN-γ, CD107a, Perforin, and Granzyme B were observed, in the effector memory (**Figure 4A**) and the central memory compartment (**Figure 4B**). Both subsets were nearly absent in VC or ART patients and correlated convincingly with VSC (**Figures 4A, B**). Interestingly a third distinct subset with expression of CXCR5 and cytotoxic markers was identified (**Figure 4C**) which was significantly more prevalent in EC compared to ART but did not correlate with VSC (**Figure 4C**). Taken together, the identified subsets suggest the importance of multiple distinct cytotoxic subsets during natural control of HIV-1.

### Slight Increase in TNF-α but No Further Differences in Cellular Phenotype nor Soluble Factors During the Co-Culture of VIA

**Figure 5:**
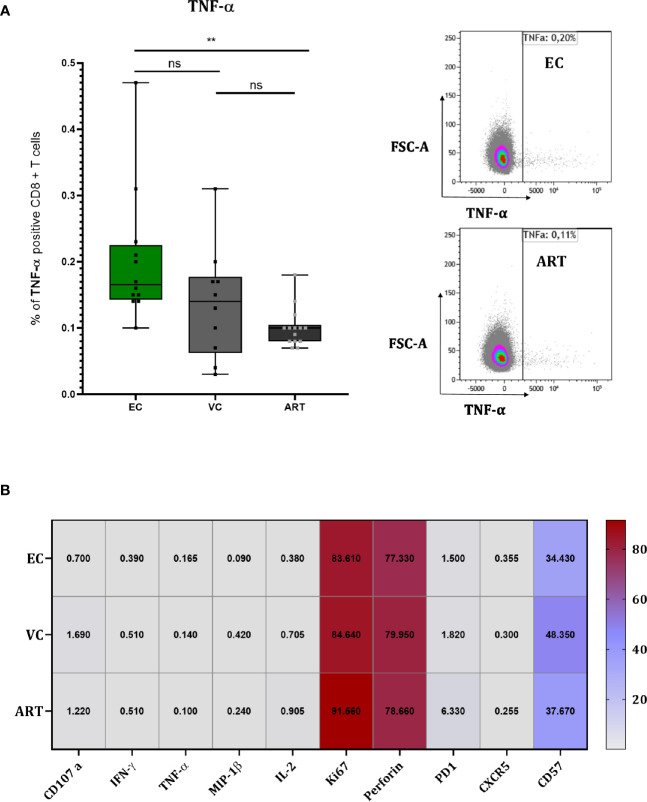
CD8^+^ T cells of EC show slightly higher TNF-α production but overall no differences during coculture screening. TNF-α measured at 120 h in coculture with representative flow charts **(A)**. Heat Map depicting median expression of proteins in columns and patient group in rows with exact values plotted inside of the cells of the heat map **(B)**. Statistical testing by Mann Whitney nonparametric statistics. **<0.01; ns, not significant.

To explore the mechanism of action by which CD8^+^ T cells suppress viral outgrowth during VIA culture we performed flow cytometry at 120 h in co-culture (**Figure 5**). TNF-α showed a slight but significant increase in EC compared to ART (**Figure 5A**) but its expression did not correlate with VSC (data not shown). For all other proteins, a strong expression of the proliferation marker (Ki67), the effector molecule (Perforin) and terminal differentiation marker (CD57) was detected in all patient groups (**Figure 5B**). Especially the high levels of Perforin suggest an involvement of direct cell to cell killing. PD-1 expression trended higher in ART patients without any significance compared to viral controllers (**Figure 5B**). Overall, besides TNF-α, single marker expression levels did not show any significant differences between patient groups. Furthermore, Boolean analysis of combinatorial expression revealed no significant differences among clinical phenotypes as well (data not shown). In addition, we assessed the effect of in-vitro superinfection of CD4^+^ T cells on the CD8^+^ T cells responses during coculture by comparing cocultures of non-superinfected CD4^+^ T cells with superinfected CD4^+^ T cells. We did not detect any differences (**Supplemental Figure 5**). Interestingly and although only assessed on a small number patients, we observed a trending difference in CD8^+^ T cells co-expressing Ki67, Perforin and IFN-γ at only 48 h in coculture (**Supplementary Figure 6**). In addition to these data we further assessed soluble markers (IFN-γ, IL-6, IP10, MIP-1β, TNF-α and TRAIL) from the culture supernatants at 72 h in coculture with no significant changes across patient groups (**Supplementary Figure 7**).

## Discussion

Evidence for the importance of anti–HIV-1 CD8^+^ T cell responses during natural control continues to substantiate. In our study, we evaluated two aspects of *in vitro* viral suppression in patients with natural control of HIV-1 infection and well-treated progressors: the *ex vivo* “effector function” (unstimulated condition) as well as the “memory function” (after *in vitro* stimulation with Gag peptides for 7 days). We focused specifically on the immune phenotypes of CD8^+^ T cells and their functionality. While VSC did only weakly correlate with the viral reservoir and not with the clinical parameters of the patients, we observed a clear association with IFN-γ secretion and proliferation markers of CD8^+^ T cells. Furthermore, we identified three cytotoxic subsets with different tissue homing abilities specific for natural control of HIV-1. One of these Gag reactive memory subsets expressed CXCR5 thus has potential access to the B cell zone of lymphoid tissues. Overall, we show that the use of a polyvalent Gag peptide pool induced a significant increase of VSC in HIV-1 infected patients, including treated progressors. However, multifunctional cytotoxic CD8 T cell responses emerge in HIV controllers only and hence might be key to sustained ART free HIV-1 control.

Several studies have reported elevated *ex vivo* VSC in EC compared to progressors providing a direct link between control of viremia and anti-viral CD8^+^ T cell immunity (9, 25, 26, 28, 38, 39). We observed a significant higher suppression of EC in non-stimulated CD8^+^ T cells as well, however with a higher heterogeneity across EC compared to other reports (**Figure 1A**). Interestingly the use of lower E:T ratio (1:10) abrogates suppression of non-stimulated CD8^+^ T cells in our setting in contrast to what Cirion et al. have reported. They observed the same level of VSC for HIV controllers in 1:1 and 1:10 ratios (38). Possible reasons for these divergent findings could be the slightly different technical setup for the VIA and different Gag peptide pools for stimulation. In addition, the used cryopreserved PBMCs could have introduced variance, although we applied the widely established procedure of overnight resting for functional studies on lymphocytes before use (40, 41), we cannot rule out an effect of cryopreservation.

In contrast with most other studies we included VC and EC as two distinctive groups of slow progressors. It has to be emphasized that the current median pVL in this group was 86 copies/ml (**Table 1**). Consequently at this low range a big proportion of these patients was categorized as EC in the past when detection limits for pVL were at 50 copies/ml or higher. Therefore, caution is advised when relating our findings to the existing literature. Secondly, most studies either do merge EC and VC or just look at EC. As a result, studies on VC are comparably scarce. Our data shows that VC excerpt comparable VSC to EC (**Figure 1A**). The study from Spentzou et al. has reported similar results (34). This suggests that slightly elevated pVLs do not reflect a loss of CD8^+^ T cell suppression. Interestingly at the current understanding elevated pVL could even contribute to the maintenance of anti-viral CD8^+^ T cell responses because decreasing pVLs during the initiation of ART associated with loss of VSC in progressors (39). But for VC we did not find any correlation between pVL and VSC (**Supplementary Figure 4**), suggesting that the level of viremia in these patients does not reflect a loss or gain in CD8^+^ T cell functionality.

In several previous studies Gag-specific CD8^+^ T cell responses (measured by ELISPOT or flow cytometry) were inversely correlated to viral load and were also described to be a major contributor to increased VSC (27, 28, 42). We sought to investigate the potential of 7 days Gag stimulation using the PTE Gag peptide pool (NIH reagents program) across the different patient groups. These peptides cover multiple overlapping sequences reflecting the natural diversity of circulating HIV-1 strains (21). Consequently, they offer a large antigenic breadth, the coverage of potential T cell epitopes, and depth, the cross recognition of variants of epitopes. Hence many of those are clustered in conserved regions (43–45). Likewise, the importance of breadth in the anti-Gag response during control of HIV-1 or SIV has been underscored (46, 47). To this end, we hypothesized that expanding such memory T cells creates an opportunity to delineate the decisive CD8^+^ T cell subsets of HIV control. This because long lasting stimulation with conserved Gag epitopes boosted VSC in progressors (30) and thus could allow to identify detailed functional differences in T cell subsets compared to controllers.

We observed that VSC increased with several orders of magnitude significantly after stimulation with PTE Gag peptides in all patient groups (**Figure 1C**). This strong effect reflects their potency possibly rooted in the rapid recognition of Gag epitopes as they can be presented on HLA-I molecules even before viral integration (48, 49). The ability to redirect CTL killing against reactivated autologous virus by Gag stimulation was reported previously for EC and ART, however using IL2 during pre-stimulation of CD8 + T cells (50), which we omitted to not alter the phenotype of cells by exogenous cytokines and reduce background noise in the VIA. Notably both controller groups showed significantly higher VSC after peptide stimulation as compared to ART (**Figure 1B**). This might be rooted in an increased immunodominance of anti-Gag T cells of controllers (51, 52), which could be further amplified by their expansion. The significantly higher expression of Ki67, a proliferation marker, on CD8^+^ T cells of EC could support this hypothesis (**Figure 3C**). Importantly the proliferation of antigen specific CD8^+^ T cells was associated to natural control by other studies as well (10, 11). Shan et al. also observed increased proliferation for ART patients in response to Gag peptides (50), our data revealed significant higher expression of Ki67 pointing into a similar direction (**Figure 3C**). Next, we saw that the VSC of EC after pre-stimulation with Gag PTE stayed elevated even when E:T ratio is decreased to 1:10 (**Figure 1B**) indicating a higher magnitude in Gag specific CD8^+^ T cell expansion (as shown by higher Ki67 expression in EC) (**Figure 3C**) or a stronger per cell suppressive capacity.

To explore this aspect in detail we applied IFN-γ ELISPOT at baseline and after 7 days of peptide stimulation. ELISPOT results clearly showed the increased frequency of IFN-γ-secreting T cells in PBMCs of EC at baseline and after prestimulation (**Figures 2A, C**). We observed only moderate correlation with VSC for both conditions (**Figures 2B, D**). Other studies reported also divergent results (28, 53). Interestingly an *in vitro* vaccination study reported a correlation with IFN-γ only when co-expressed with CD107a and MIP-1β (54). This suggests that not IFN-γ alone but rather multifunctionality might be the best reflector of increased VSC.

To explore functionality and phenotype on a single cell level we phenotyped by 18 color flow cytometry without and after 7 days of peptide stimulation. The expression of IFN-γ in total CD8^+^ T cells was significantly increased only after stimulation between EC and VC and ART (**Figure 3A**). The degranulation marker CD107a increased significantly for both groups, VC and EC (**Figure 3B**). Interestingly Ki67 expression increased significantly in all three groups which was suggestive of a specific HIV-1 proliferative response to the Gag epitopes (**Figure 3C**), and corroborated by its strict absence in HD controls (**Figures 3A–C**). Ki67 did show moderate correlation with viral suppression *in vitro*.

Polyfunctional anti-viral CD8^+^ T cells, defined as “having the ability to secrete chemokines and mediate cytolysis” (55), are a qualitative feature of EC (18, 45, 56). To assess the combinatorial expression of functional and phenotypic we applied a Boolean gating strategy. By doing so, we identified three distinct subsets significantly increased in EC. Two of those showed strong cytotoxic characteristics (**Figures 4A, B**) from the EM and CM compartment which correlated convincingly with VSC (**Figure 4**). This provides evidence for the co-existence of multiple subsets that could be implicated in HIV-1 control homing to central lymphoid organs and peripheral tissues.

Intriguingly Gag peptide stimulation upregulated CXCR5 in EC and ART patients (**Figure 3D**). Recent studies suggest that circulating CXCR5^+^ CD8^+^ T cells are major producers of IL21 and associate with limited HIV-1 replication (57). We cannot confirm differences either at baseline nor after peptide stimulation. However a cytotoxic subset expressing CXCR5 with potential access to the B cell follicles (**Figure 4C**) a major site of the viral reservoir (58, 59) was significantly expanded in natural controllers. Recent literature is controversial on the functionality of these cells. Reuter et al. have reported a strong correlation between CXCR5+ CD8^+^ T cells isolated from lymph node (LN) and pVL in VC but a decreased killing ability compared to bulk CD8^+^ T cells from PBMCs of the same patients (60). In contrast, although Petrovas et al. observed a similar strong correlation with pVL they find increased killing capacities of follicular CD8^+^ T cells from LN as compared to CXCR5 negative counterparts despite their compromised cytokine profile (61). To our knowledge, we characterized for the first time a polyfunctional CD8^+^ T cell subset combining features of cytotoxicity, proliferation and expressing CXCR5 after expansion with Gag peptides. However we observed no correlation with VSC (**Figure 4C**) that could be explained by the low presence of this subset in blood. Still, this finding is of utmost importance since follicular helper CD4^+^ T cells (TFH), residing in B-cell follicles of secondary lymphoid tissues, represent a major site of viral persistence (58). Future studies need to evaluate if Gag specific CXCR5 positive subsets have suppressive abilities and access to the LN of HIV controllers. Importantly, a recent study has further substantiated that the predominant proportion of circulating TCR repertoires in HIV-1 infected patients under ART is able to target latent reservoirs (62). Future studies now should scrutinize how to achieve proper qualitative features in such effector cells. In conclusion, therapies boosting existing T cell immunity are a potential angle of intervention but need to prove their potency *in vivo* as resistance of reservoirs to complete elimination was a concern raised earlier (63).

To elucidate the mechanism of HIV-1 CD8^+^ T cell mediated suppression *in vitro* during the VIA coculture we phenotyped T cells 120 h after the coculture when solid viral replication was observed by intracellular P24 staining (data not shown). Only for TNF-α we found a very minor increase which didn’t correlate with VSC. Overall we could not identify any clear significant differences between patient groups, but the strong increase of Perforin on total CD8^+^ T cells of all patients (**Figure 5B**) suggests a direct cytotoxic mechanism as previously proposed (9). Interestingly we detected Perforin, IFN-γ and Ki67 positive subsets at 48 h between EC and ART (**Supplementary Figure 6**) in a subset of seven patients suggesting that the assessment for phenotypic and functional properties should be done earlier, before notable viral replication. Furthermore, at 120 h in coculture the effect of exogenous IL-2 supplemented to the VIA media might have already levelled out phenotypic differences between clinical phenotypes. A similar effect might apply to the levels of cytokines or chemokines at 72 h in coculture (**Supplementary Figure 7**). Overall the exact mechanism of viral suppression *in vitro* remains to be determined.

In conclusion, our work demonstrated that stimulation with polyvalent Gag peptide pools efficiently increased VSC in all patient groups. Interestingly upregulation of CXCR5 expression was noted upon pre-stimulation. The increased frequencies of several distinct multifunctional CD8^+^ T cells subsets which correlated convincingly with the magnitude of viral suppression is an particular attribute of natural controllers. Furthermore Gag targeting CXCR5^+^CD8^+^ T cells represent a unique subset of antiviral CD8^+^ T cells that may play a pivotal role in the containment of reservoirs. Overall, our data highlighted a multi-qualitative anti-Gag CD8^+^ T cell response during control of HIV-1 that should be elicited for future vaccination strategies to achieve ART free viral remission.

## Data Availability Statement

The raw data supporting the conclusions of this article will be made available by the authors, without undue reservation.

## Ethics Statement

Blood samples were collected in accordance with the Declaration of Helsinki. The study was approved by each local ethical committee and received the ethical approval from the university hospital of Antwerp (Belgium) under the registration number: B300201731330 as a multicenter Belgian cohort called “PhenoCure”. All participants gave written informed consent.

## PhenoCure Study Group

The authors of the PhenoCure Study group are: Eric Florence, eflorence@itg.be, Institute of tropical medicine, HIV/STD outpatient department, Antwerp Belgium; Maartje van Frankenhujizen, mvanfrankenhuijsen@itg.be, Institute of tropical medicine, HIV/STD outpatient department, Antwerp Belgium; Joeri Aerts, joeri.Aerts@vub.be, Vrije Universiteit Brussel, Neuro-aging and Viro-imunotherapy research group, Brussels, Belgium; Sabine Allard, Sabine.Allard@vub.ac.be, Universitair Ziekenhuis Brussels, internal medicine-endocrinology, Brussels, Belgium; Stephane De Wit, stephane.de.wit@ulb.ac, Saint-Pierre University Hospital, Service of Infectious Diseases, Brussels, Belgium; Nescoi Coca, coca.necsoi@stpierre-bru.be, Saint-Pierre University Hospital, Service of Infectious Diseases, Brussels; Peter Messiaen, peter.messiaen@uhasselt.be, Jessa Ziekenhuis, Hasselt, Belgium; Michel Moutschen, mmoutschen@chu.ulg.ac.be, Liège University Hospital, Université de Liège, Liège, Belgium.

## Author Contributions

PA designed, performed and analyzed experiments, drafted figures, and wrote the manuscript. GI did setup some of the used FACS panels. J-YS did the multiplex ELISA and supported with P24 ELISA experiments. LV did perform total HIV-1 DNA measurements by ddPCR. GV designed and supervised the study and wrote the manuscript. CS-D designed and supervised the study. The PhenoCure Study group designed and performed the recruitment of the patients and the collection of the samples. All authors contributed to the article and approved the submitted version.

## Funding

PA received an AFR PhD grant (ID: PHD-2015-1-10111126) from the FNR in Luxembourg.

## Conflict of Interest

The authors declare that the research was conducted in the absence of any commercial or financial relationships that could be construed as a potential conflict of interest.

## Supplementary Material

Supplementary Figure 1 IL18 concentration in plasma differs significantly between EC and ART. Plasma samples isolated during blood donation were analyzed for the concentration of cytokines IL18 **(A)**, IL-6 **(B)**, IP-10 **(C)**, and TNF-α **(D)**. Statistical testing by nonparametric Kruskal Wallis test with Dunn correction for multiple comparison.

Supplementary Figure 2 HIV-1 DNA is significantly lower in EC compared to ART and has a weak negative correlation with VSC over the whole cohort. Absolute quantification of cell associated HIV-1 DNA on purified CD4 + T cells by ddPCR. Five samples **(A)** (two of EC and three of VC measurements were below the confidence interval of the negative control and cannot be accurately quantified. These values have been excluded from the analysis to preclude statistical bias). Testing was done with Kruskal Wallis nonparametric statistics with Dunn correction. Spearman correlation of cell associated HIV-1 DNA with VSC on non-stimulated **(B)** and stimulated **(C)** cells.

Supplementary Figure 3 No correlation with IFN-γ, CD107a, CXCR5 but a weak correlation with Ki67 expression and VSC. Correlation of CD8^+^ T cell expression of IFN-γ **(A)**, CD107a **(B)**, Ki67 **(C)**, and CXCR5 **(D)** with VSC. Non-parametric rank spearman correlation with rho and p values are given in the figure.

Supplementary Figure 4 No correlation of pVL with VSC of VC. Spearman correlation of pVL with VSC for non-stimulated **(A)** and stimulated **(B)** CD8^+^ T cells given for VC only because all other patients of the cohort have undetectable viremia. Statistical values plotted in the graph.

Supplementary Figure 5 No impact on the cellular phenotypes detected by superinfection at 120 h in coculture. To assess if the superinfection with HIV-1 (III_B_) has a detectable impact on the CD8^+^ T cell phenotypes we analyzed superinfected and non-superinfected conditions separately by flow cytometry. No differences were found as exemplified here for IFN-y **(A)** and CD107a **(B)** staining’s. Statistics by paired Mann Whitney nonparametric testing.

Supplementary Figure 6 A trend between EC and ART patients of Ki67, Perforin, and IFN-γ co-expressing CD8^+^ T cells at 48 h in coculture. To explore the kinetic pattern of cytotoxic markers during coculture we phenotyped seven patients at 48 h. Statistical testing by Mann-Whitney nonparametric comparison.

Supplementary Figure 7 Concentration of cytokines at 3 days in VIA coculture revealed no differences between patient groups. Supernatants of VIA coculture at 72 h (day 3) were tested with mutliplex ELISA for IFN-γ **(A)**, IL-6 **(B)**, IP-10 **(C)**, MIP-1β **(D)**, TNF-α **(E)**, and TRAIL **(F)**. Statistical testing by nonparametric Kruskal Wallis test with Dunn correction for multiple comparison.
