# Supplementary figures and images for: Cytotoxic CD8+ T Cells Expressing CXCR5 Are Detectable in HIV-1 Elite Controllers After Prolonged In Vitro Peptide Stimulation
Source: Front Immunol. 2021 Feb 24;11:622343. doi: 10.3389/fimmu.2020.622343 (PMC7945035; doi:10.3389/fimmu.2020.622343)

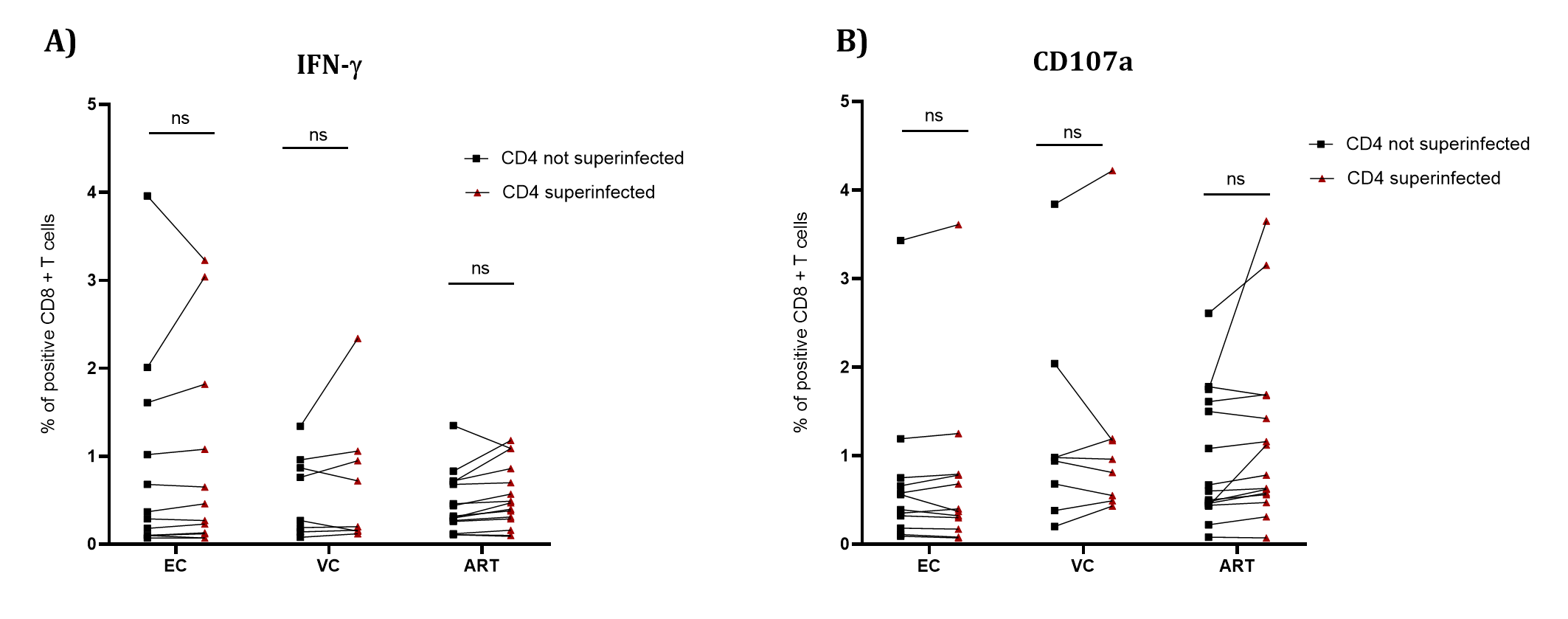

Supplement: Supplementary Figure 1 — IL18 concentration in plasma differs significantly between EC and ART. Plasma samples isolated during blood donation were analyzed for the concentration of cytokines IL18 (A), IL-6 (B), IP-10 (C), and TNF-α (D). Statistical testing by nonparametric Kruskal Wallis test with Dunn correction for multiple comparison. [file Image_1.tif]

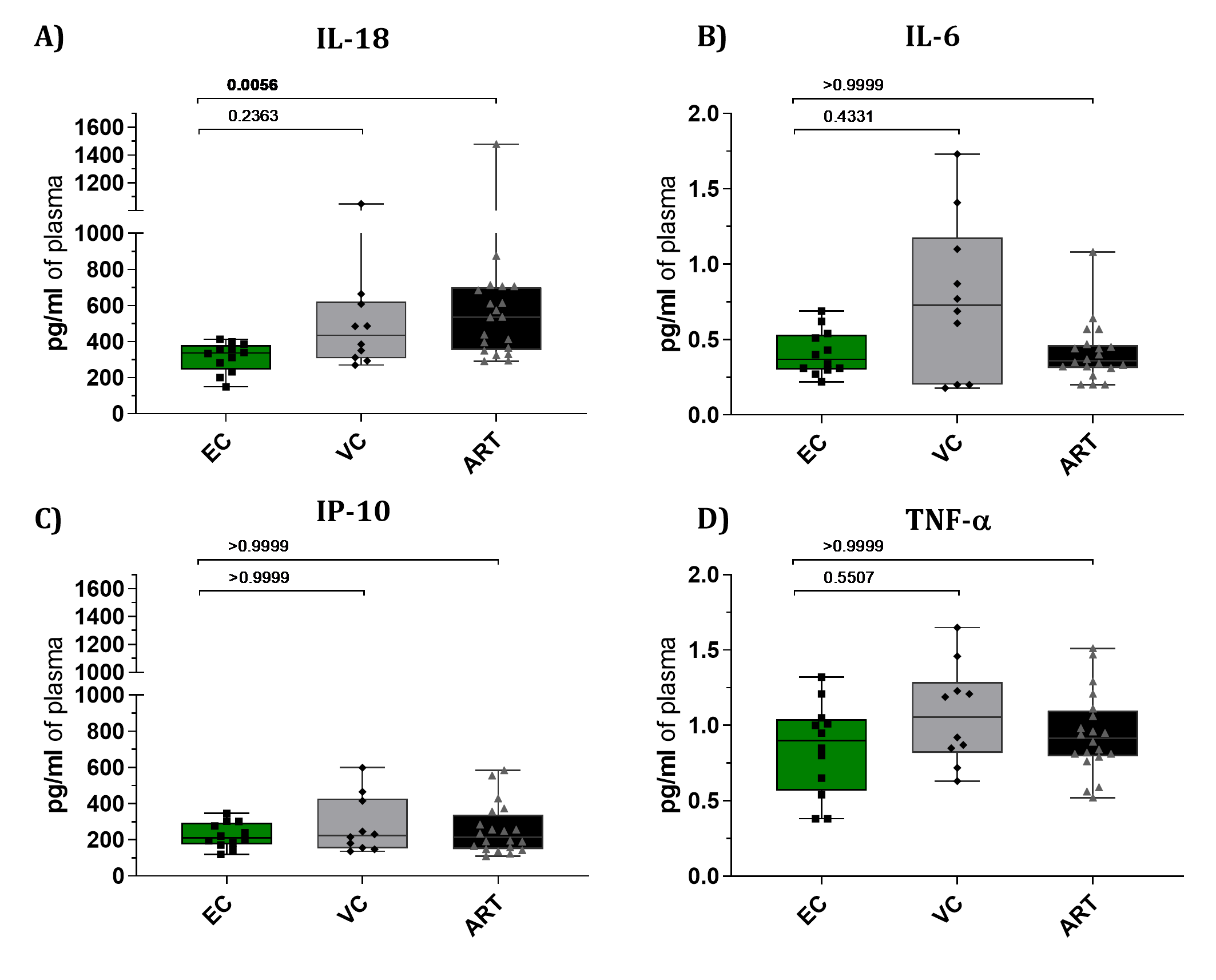

Supplement: Supplementary Figure 2 — HIV-1 DNA is significantly lower in EC compared to ART and has a weak negative correlation with VSC over the whole cohort. Absolute quantification of cell associated HIV-1 DNA on purified CD4 + T cells by ddPCR. Five samples (A) (two of EC and three of VC measurements were below the confidence interval of the negative control and cannot be accurately quantified. These values have been excluded from the analysis to preclude statistical bias). Testing was done with Kruskal Wallis nonparametric statistics with Dunn correction. Spearman correlation of cell associated HIV-1 DNA with VSC on non-stimulated (B) and stimulated (C) cells. [file Image_2.tif]

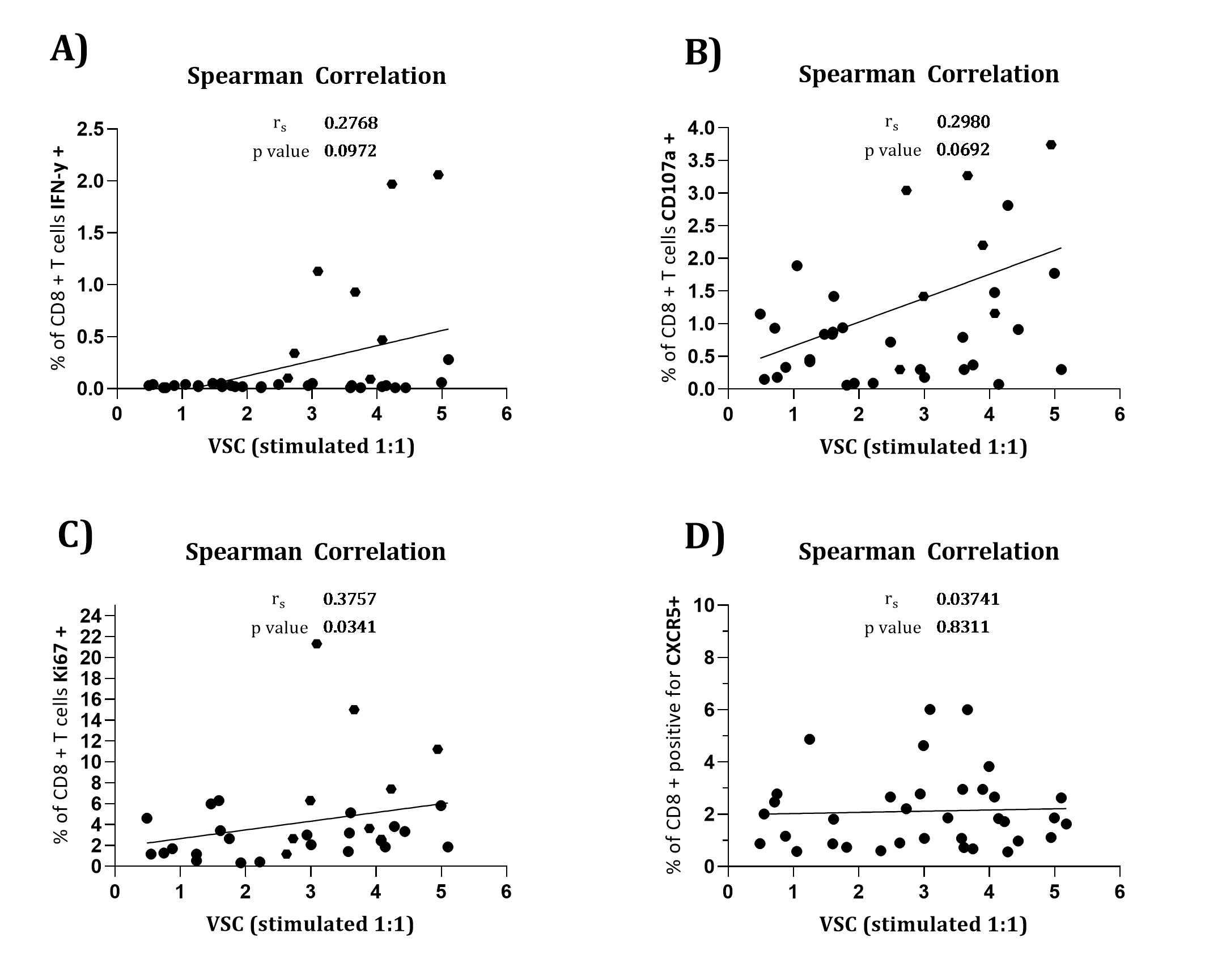

Supplement: Supplementary Figure 3 — No correlation with IFN-γ, CD107a, CXCR5 but a weak correlation with Ki67 expression and VSC. Correlation of CD8+ T cell expression of IFN-γ (A), CD107a (B), Ki67 (C), and CXCR5 (D) with VSC. Non-parametric rank spearman correlation with rho and p values are given in the figure. [file Image_3.tif]

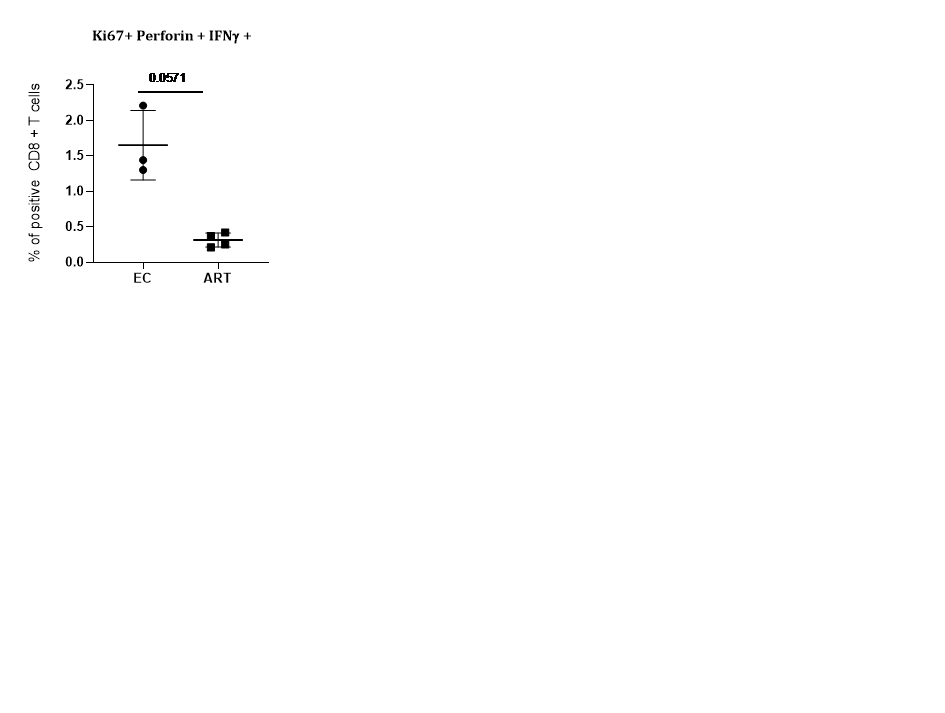

Supplement: Supplementary Figure 4 — No correlation of pVL with VSC of VC. Spearman correlation of pVL with VSC for non-stimulated (A) and stimulated (B) CD8+ T cells given for VC only because all other patients of the cohort have undetectable viremia. Statistical values plotted in the graph. [file Image_4.tif]

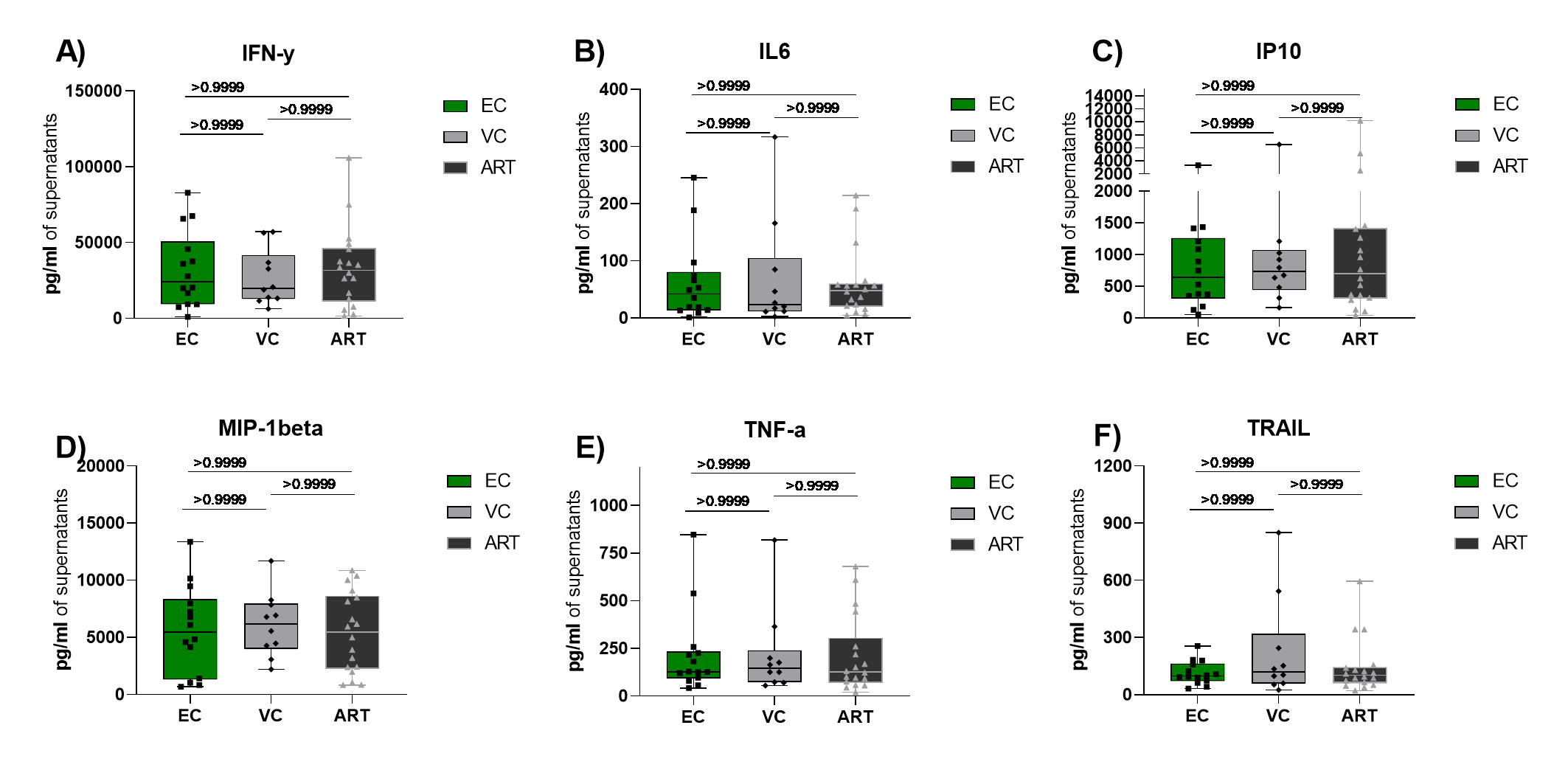

Supplement: Supplementary Figure 5 — No impact on the cellular phenotypes detected by superinfection at 120 h in coculture. To assess if the superinfection with HIV-1 (IIIB) has a detectable impact on the CD8+ T cell phenotypes we analyzed superinfected and non-superinfected conditions separately by flow cytometry. No differences were found as exemplified here for IFN-y (A) and CD107a (B) staining’s. Statistics by paired Mann Whitney nonparametric testing. [file Image_5.tif]

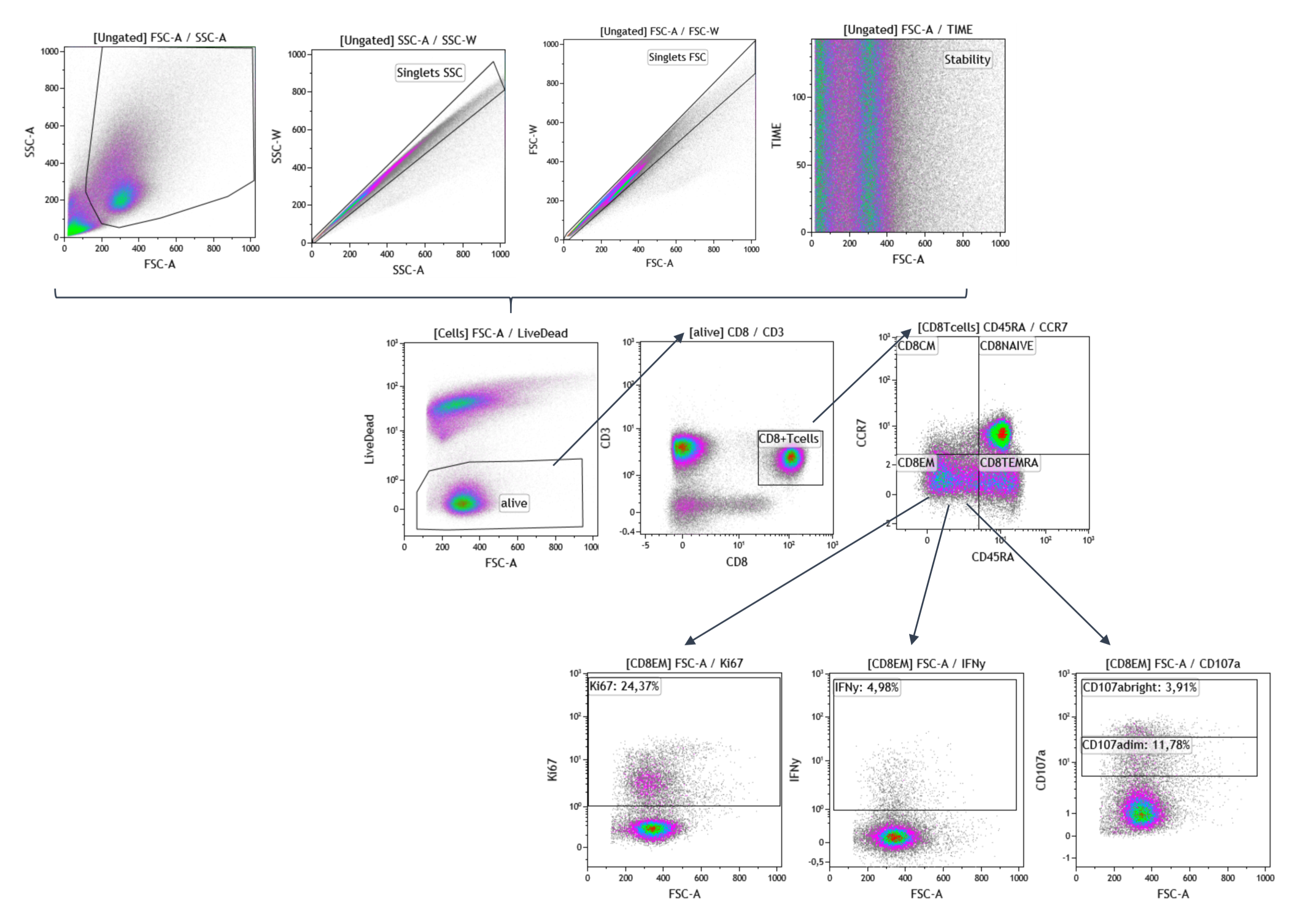

Supplement: Supplementary Figure 6 — A trend between EC and ART patients of Ki67, Perforin, and IFN-γ co-expressing CD8+ T cells at 48 h in coculture. To explore the kinetic pattern of cytotoxic markers during coculture we phenotyped seven patients at 48 h. Statistical testing by Mann-Whitney nonparametric comparison. [file Image_6.tif]

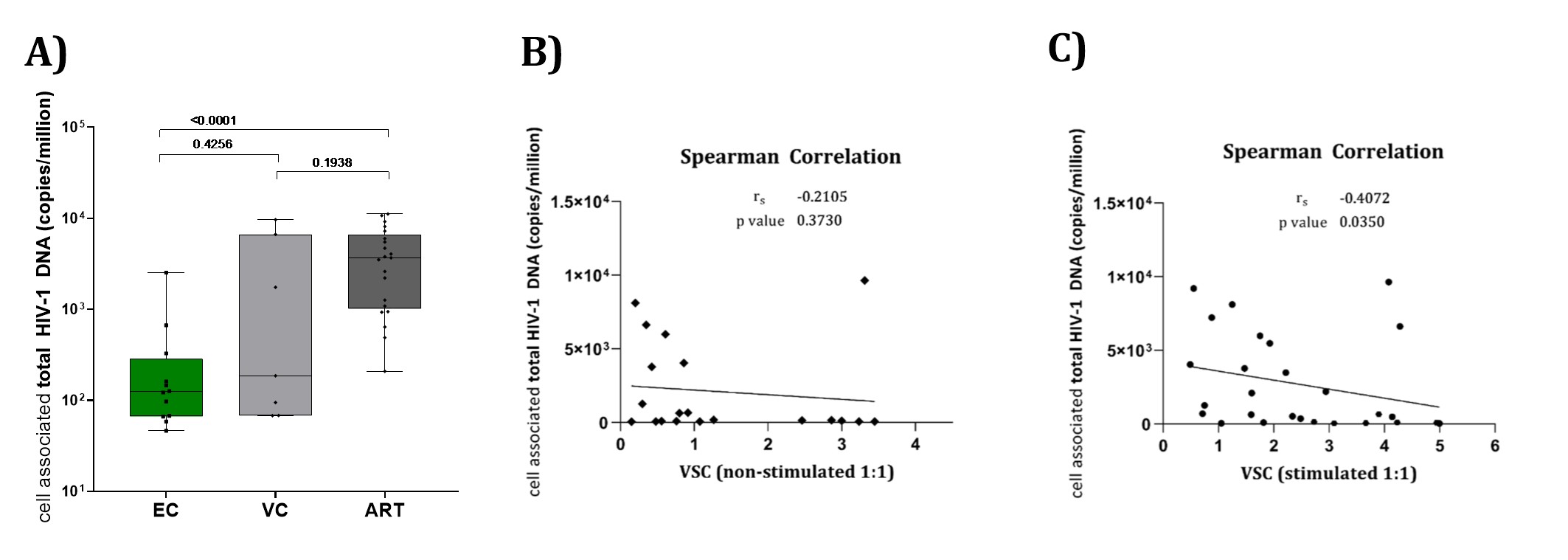

Supplement: Supplementary Figure 7 — Concentration of cytokines at 3 days in VIA coculture revealed no differences between patient groups. Supernatants of VIA coculture at 72 h (day 3) were tested with mutliplex ELISA for IFN-γ (A), IL-6 (B), IP-10 (C), MIP-1β (D), TNF-α (E), and TRAIL (F). Statistical testing by nonparametric Kruskal Wallis test with Dunn correction for multiple comparison. [file Image_7.tif]
