# Supplementary material for: Cytotoxic CD8+ T Cells Expressing CXCR5 Are Detectable in HIV-1 Elite Controllers After Prolonged In Vitro Peptide Stimulation
Source: Front Immunol. 2021 Feb 24;11:622343. doi: 10.3389/fimmu.2020.622343 (PMC7945035; doi:10.3389/fimmu.2020.622343)
Supplement: Supplementary file 8 [file DataSheet_1.zip › Information sheet on p24ELISA analysis.pdf]

1) <u>General organization:</u>

Each run of viral inhibition assays (VIA) contained several randomly selected patients with at least one of each clinical category (Elite controller (EC), Viral controller (VC), ART treated progressor (ART)). The final data was generated from seven runs. Consequently the raw data is organized in seven folders, one per VIA.

2) <u>Labelling of the data files:</u>

For identification, raw data files (.xls) were named as explained by the following example (these different identifiers are important to properly assign the measurement per patients, as explained in the next section).

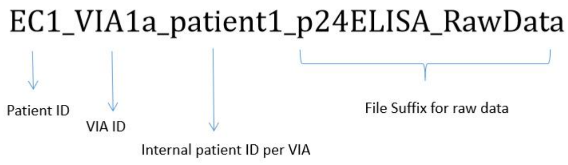

3) <u>ELISA layouts:</u>

Culture supernatans from patients was assessed with P24 ELISA (BIOMARIC) using seven 1:5 serial dilutions starting from 1:20 (see below figure 1 A-B, dilution factors in the left column). Positive controls, plate controls, negative controls and blanks were run on each ELISA plate (see labels). NIBSC p24 standards were diluted from 10 IU/ml (equalling 217 pg/ml on BIOMARIC p24ELISA determined by the company) in 1:2 serial dilutions (see below, highlighted in yellow) on every second plate per run. For each patient five conditions were assessed (each in triplicate as described in detail in the article):

1) overnight 1:1 2) overnight 1:10 3) PTE 1:1 4) PTE 1:10 5) CD4 only

Consequently for each patient one ELISA plate was run in layout 1 (figure 1, A) and then four patients in layout 2 (figure 1, B)

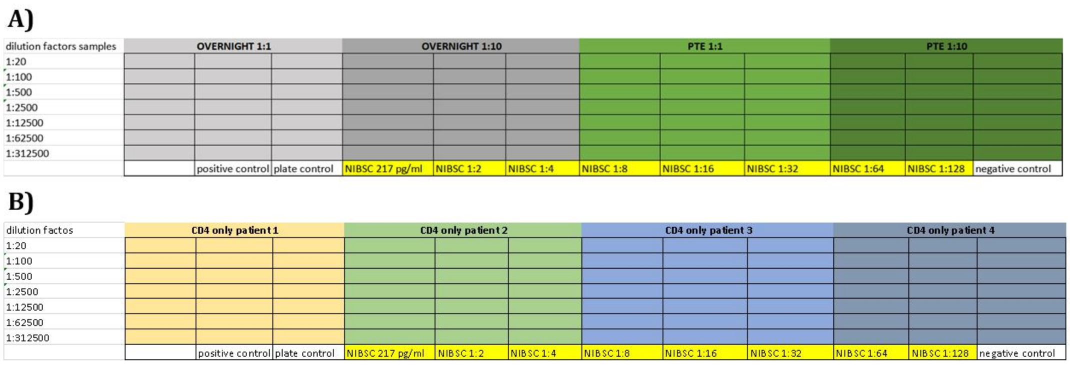

Figure 1: Layout of ELISA plates. For each patient a full plate containing conditions 1-4 (A). CD4 T cell only conditions were run for four patients per plate coming from the same VIA run (B).

4) <u>Data analysis:</u>

P24 concentrations were determined as follows

A) Optical density (OD) at 450nm was subtracted from 605 nm values

B) Standard curve was calculated using all standard curves determined in each VIA ELISA run

C) Concentration of P24 in culture supernatans was determined by linear interpolation to the standard curve. Then averages were calculated from triplicates per condition(s) and multiplied by the respective dilution factors to obtain the p24 concentration in pg/ml.

D) Lastly viral inhibitory capacity (VSC) was determined by subtracting the respective condition from the CD4 only outgrowth in log10 (as explained in the material and method section).

Find a full example calculation below:

Viral inhibition assay - Metadata and Analysis information

VSC calculation EC1_VIA1a:

Test Name: P24BIOMARIC — Date: 16.07.2019 — Time: 14:27:05

ID1: 160719_plate1_VIA1

Absorbance — Absorbance values are displayed as OD

1. Raw Data (450 1)

	1	2	3	4	5	6	7	8	9	10	11	12
**A**	3,5	3,5	3,5	3,5	3,5	3,5	0,222	0,221	0,196	2,934	3,5	1,061
**B**	3,5	3,5	3,5	3,5	3,5	3,5	0,11	0,104	0,109	1,215	3,074	0,305
**C**	3,5	3,5	3,5	3,5	3,5	3,5	0,099	0,085	0,08	0,371	1,234	0,137
**D**	3,398	3,5	3,5	3,355	3,5	3,5	0,082	0,082	0,078	0,148	0,392	0,105
**E**	1,667	1,859	1,788	1,574	2,119	1,856	0,078	0,08	0,077	0,092	0,157	0,1
**F**	0,645	0,744	0,719	0,693	0,881	0,78	0,091	0,081	0,082	0,091	0,111	0,098
**G**	0,258	0,266	0,272	0,253	0,341	0,29	0,086	0,079	0,074	0,086	0,085	0,113
**H**	0,087	3,013	1,01	2,523	1,738	1,031	0,61	0,386	0,24	0,171	0,134	0,115

2. Raw Data (605 2)

	1	2	3	4	5	6	7	8	9	10	11	12
**A**	0,062	0,074	0,084	0,05	0,052	0,051	0,039	0,031	0,028	0,032	0,035	0,031
**B**	0,037	0,031	0,041	0,037	0,039	0,041	0,033	0,029	0,031	0,03	0,032	0,039
**C**	0,03	0,029	0,036	0,035	0,037	0,05	0,029	0,029	0,029	0,031	0,031	0,029
**D**	0,035	0,034	0,034	0,026	0,033	0,034	0,032	0,03	0,029	0,03	0,03	0,031
**E**	0,031	0,029	0,031	0,028	0,032	0,029	0,03	0,028	0,029	0,025	0,027	0,032
**F**	0,025	0,037	0,034	0,026	0,031	0,028	0,029	0,027	0,029	0,03	0,031	0,029
**G**	0,03	0,03	0,03	0,026	0,031	0,028	0,032	0,027	0,028	0,029	0,026	0,041
**H**	0,03	0,031	0,031	0,029	0,032	0,028	0,032	0,028	0,028	0,029	0,029	0,029

1) OD450 - OD605

	1	2	3	4	5	6	7	8	9	10	11	12
**A**	3,438	3,426	3,416	3,45	3,448	3,449	0,183	0,19	0,168	2,902	3,465	1,03
**B**	3,463	3,469	3,459	3,463	3,461	3,459	0,077	0,075	0,078	1,185	3,042	0,266
**C**	3,47	3,471	3,464	3,465	3,463	3,45	0,07	0,056	0,051	0,34	1,203	0,108
**D**	3,363	3,466	3,466	3,329	3,467	3,466	0,05	0,052	0,049	0,118	0,362	0,074
**E**	1,636	1,83	1,757	1,546	2,087	1,827	0,048	0,052	0,048	0,067	0,13	0,068
**F**	0,62	0,707	0,685	0,667	0,85	0,752	0,062	0,054	0,053	0,061	0,08	0,069
**G**	0,228	0,236	0,242	0,227	0,31	0,262	0,054	0,052	0,046	0,057	0,059	0,072
**H**	0,057	2,982	0,979	2,494	1,706	1,003	0,578	0,358	0,212	0,142	0,105	0,086

2) Standard curve

NIBSC concentration (pg/ml)	217	108,5	54,25	27,125	13,5625	6,78125	3,390625	1,6953125
OD measured	2,413	1,531	0,958	0,591	0,331	0,201	0,155	0,13
	2,494	1,706	1,003	0,578	0,358	0,212	0,142	0,105
Average OD	2,410333333	1,600333333	0,969	0,581333333	0,35	0,210333333	0,148333333	0,109

Create formula from the linear range of the dilutions

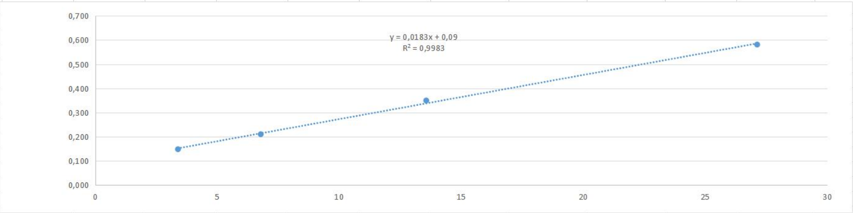

Assess values in the linear portion of the plate:

	1	2	3	4	5	6	7	8	9	10	11	12
**A**	3,438	3,426	3,416	3,45	3,448	3,449	0,183	0,19	0,168	2,902	3,465	1,03
**B**	3,463	3,469	3,459	3,463	3,461	3,459	0,077	0,075	0,078	1,185	3,042	0,266
**C**	3,47	3,471	3,464	3,465	3,463	3,45	0,07	0,056	0,051	0,34	1,203	0,108
**D**	3,363	3,466	3,466	3,329	3,467	3,466	0,05	0,052	0,049	0,118	0,362	0,074
**E**	1,636	1,83	1,757	1,546	2,087	1,827	0,048	0,052	0,048	0,067	0,13	0,068
**F**	0,62	0,707	0,685	0,667	0,85	0,752	0,062	0,054	0,053	0,061	0,08	0,069
**G**	0,228	0,236	0,242	0,227	0,31	0,262	0,054	0,052	0,046	0,057	0,059	0,072
**H**												

Interpolation

y = ax + b	a =	0,0269
x = (y - b) / a	b =	0,084

	1	2	3	4	5	6	7	8	9	10	11	12
**A**	124,6840149	124,2379182	123,866171	125,1301115	125,0557621	125,0929368	3,680297398	3,940520446	3,12267658	104,7583643	125,6877323	35,16728625
**B**	125,6133829	125,8364312	125,464684	125,6133829	125,5390335	125,464684	-0,260223048	-0,334572491	-0,223048327	40,92936803	109,9628253	6,765799257
**C**	125,8736059	125,9107807	125,6505576	125,6877323	125,6133829	125,1301115	-0,520446097	-1,040892193	-1,226765799	9,516728625	41,59851301	0,892193309
**D**	121,8959108	125,7249071	125,7249071	120,6319703	125,7620818	125,7249071	-1,26394052	-1,189591078	-1,301115242	1,26394052	10,33457249	-0,371747212
**E**	57,69516729	64,9070632	62,19330855	54,34944238	74,46096654	64,79553903	-1,338289963	-1,189591078	-1,338289963	-0,63197026	1,710037175	-0,594795539
**F**	19,92565056	23,1598513	22,34200743	21,67286245	28,47583643	24,83271375	-0,817843866	-1,115241636	-1,152416357	-0,855018587	-0,148698885	-0,557620818
**G**	5,353159851	5,650557621	5,873605948	5,31598513	8,401486989	6,617100372	-1,115241636	-1,189591078	-1,412639405	-1,003717472	-0,92936803	-0,446096654
**H**												

Interpolated values multiplied by dilution factors:

	Patient 1	Patient 1	Patient 1	Patient 1	Patient 1	Patient 1	Patient 1	Patient 1	Patient 1	Patient 1	Patient 1	Patient 1
	OVNR	1/1		OVNR	1/10		PTE	1/1		PTE	1/10	
20	2493,680297	2484,758364	2477,32342	2502,60223	2501,115242	2501,858736	73,60594796	78,81040892	62,4535316	2095,167286	2513,754647	703,3457249
100	12561,33829	12583,64312	12546,4684	12561,33829	12553,90335	12546,4684	-26,02230483	-33,45724907	-22,30483271	4092,936803	10996,28253	676,5799257
500	62936,80297	62955,39033	62825,27881	62843,86617	62806,69145	62565,05576	-260,2230483	-520,4460967	-613,3828996	4758,364312	20799,25651	446,0966543
2500	304739,777	314312,2677	314312,2677	301579,9257	314405,2045	314312,2677	-3159,851301	-2973,977695	-3252,788104	3159,851301	25836,43123	-929,3680297
12500	721189,5911	811338,29	777416,3569	679368,0297	930762,0818	809944,2379	-16728,62454	-14869,88848	-16728,62454	-7899,628253	21375,46468	-7434,944238
62500	1245353,16	1447490,706	1396375,465	1354553,903	1779739,777	1552044,61	-51115,24164	-69702,60223	-72026,0223	-53438,66171	-9293,680297	-34851,30112
312500	1672862,454	1765799,257	1835501,859	1661245,353	2625464,684	2067843,866	-348513,0112	-371747,2119	-441449,8141	-313661,71	-290427,5093	-139405,2045
	0	0	0	0	0	0	0	0	0	0	0	0

Concentration:	1758054,523	2118184,634	71,62329616	10423,79182
in Log	6,24503234	6,325963813	1,855054304	4,018025729
	OVNR 1/1	OVNR 1/10	PTE 1:1	PTE 1/10
VSC:	0,555398108	0,474466635	4,945376144	2,782404719
CD4 only patient 1 (calculated from CD4 only plate):	6,800430448	6,800430448	6,800430448	6,800430448

Viral inhibition assay - Metadata and Analysis information
